# Non-linear dynamical signal characterization for prediction of defibrillation success through machine learning

**DOI:** 10.1186/1472-6947-12-116

**Published:** 2012-10-15

**Authors:** Sharad Shandilya, Kevin Ward, Michael Kurz, Kayvan Najarian

**Affiliations:** 1 Department of Computer Science, Virginia Commonwealth University, VCU Reanimation Engineering Science Center, 1818 Providence Creek Cir, Richmond, VA, 23236, MI; 2 University of Michigan, Michigan Center for Integrative Research in Critical Care, Ann Arbor, MI; 3 Department of Emergency Medicine, Virginia Commonwealth University, VCU Reanimation Engineering Science Center, Richmond, USA; 4 Department of Computer Science, Virginia Commonwealth University, VCU Reanimation Engineering Science Center, Richmond, USA

**Keywords:** Cardiac arrest, Resuscitation, Ventricular fibrillation, CPR, Defibrillation success, Shock outcome, Complex wavelet transform, Non-linear analysis, Time-series analysis, Signal decomposition, Feature selection

## Abstract

**Background:**

Ventricular Fibrillation (VF) is a common presenting dysrhythmia in the setting of cardiac arrest whose main treatment is defibrillation through direct current countershock to achieve return of spontaneous circulation. However, often defibrillation is unsuccessful and may even lead to the transition of VF to more nefarious rhythms such as asystole or pulseless electrical activity. Multiple methods have been proposed for predicting defibrillation success based on examination of the VF waveform. To date, however, no analytical technique has been widely accepted. We developed a unique approach of computational VF waveform analysis, with and without addition of the signal of end-tidal carbon dioxide (PetCO2), using advanced machine learning algorithms. We compare these results with those obtained using the Amplitude Spectral Area (AMSA) technique.

**Methods:**

A total of 90 pre-countershock ECG signals were analyzed form an accessible preshosptial cardiac arrest database. A unified predictive model, based on signal processing and machine learning, was developed with time-series and dual-tree complex wavelet transform features. Upon selection of correlated variables, a parametrically optimized support vector machine (SVM) model was trained for predicting outcomes on the test sets. Training and testing was performed with nested 10-fold cross validation and 6–10 features for each test fold.

**Results:**

The integrative model performs real-time, short-term (7.8 second) analysis of the Electrocardiogram (ECG). For a total of 90 signals, 34 successful and 56 unsuccessful defibrillations were classified with an average Accuracy and Receiver Operator Characteristic (ROC) Area Under the Curve (AUC) of 82.2% and 85%, respectively. Incorporation of the end-tidal carbon dioxide signal boosted Accuracy and ROC AUC to 83.3% and 93.8%, respectively, for a smaller dataset containing 48 signals. VF analysis using AMSA resulted in accuracy and ROC AUC of 64.6% and 60.9%, respectively.

**Conclusion:**

We report the development and first-use of a nontraditional non-linear method of analyzing the VF ECG signal, yielding high predictive accuracies of defibrillation success. Furthermore, incorporation of features from the PetCO2 signal noticeably increased model robustness. These predictive capabilities should further improve with the availability of a larger database.

## Background

Sudden cardiac death is a significant public health concern and a leading cause of death in many parts of the world [1]. In the United States, cardiac arrest claims greater than 300,000 lives annually. Survival rates for out-of-hospital cardiac arrest remain dismal [2]. Ventricular Fibrillation (VF) is the initially encountered arrhythmia in 20-30% of cardiac arrest cases [3]. Multiple reentrant circuits contribute to the VF waveform causing its pathophysiology to be extremely dynamic. A victim’s chances of survival worsen by 10% for every minute of VF that remains untreated [4].

Defibrillation is a procedure that delivers an electrical current that depolarizes a critical mass of the myocardium simultaneously. Defibrillation increases the possibility of the sino-atrial node regaining control of the rhythm. Coronary artery perfusion provided by cardio-pulmonary resuscitation (CPR) prior to defibrillation has been shown to improve chances for ROSC [4]. As victims enter the circulatory phase of cardiac arrest, predicting defibrillation success may become paramount to prevent unnecessary interruptions to CPR [5]. Repetitive unsuccessful shocks can reduce chest compression time and can cause injury to cardiac tissue, impacting heart function upon survival. Even worse, unsuccessful shocks can cause VF to deteriorate into asystole or pulseless electrical activity (PEA), which are more difficult to resuscitate [6].

The effect of acute ischemia on tissue excitability induces conversion of VF from type-1 coarse VF to type-2 smooth VF [7]. Type 1 VF has now been correlated with the multiple-wavelet theory, while type 2 has been shown to be driven by a mother rotor [8]. This conversion *partially* conforms to rapidly attenuating chances of survival with increasing VF duration [9], and can be quantified by any measure that can account for both, a decrease in amplitude and a shift in spectral composition of the signal. Fourier Transform (FT) based measures [10] assume a linear, deterministic basis for the signals, and may prove to be impracticable. Other methods [6,11,12], with somewhat more feasible definitions of post-shock success, have focused on extracting features based on the *real* Discrete Wavelet Transform (DWT). While wavelet decomposition has proven to be more effective, clinical transition of such approaches has been precluded due to low specificities.

Gundersen and colleagues [13] have shown that predictive features of the VF waveform suffer from random effects, with p-values less than 10^-3^. This was proven with a mixed effects logistic regression model. Random effect-sizes, calculated as standard deviation of the ‘random’ term in the model, varied from 73% to 189% of the feature effect-sizes. Thus an additional objective of our work aims at countering the variance due to such effects. We also hypothesized that other physiologic signals obtained during CPR, such as partial end-tidal carbon dioxide (PetCO2), can help build a more ‘complete’ model. PetCO2 monitoring allows for the measurement of exhaled carbon dioxide from a patient. The level of exhaled carbon dioxide has been positively correlated with the amount of blood flow produced by chest compressions during CPR (see Discussion).

## Data

The study was approved by Virginia Commonwealth University Institutional Review Board. Patient de-identified (personal information removed) cardiac arrest data, for a total of 57 out-of-hospital cardiac arrest (OHCA) subjects was provided by the Richmond Ambulance Authority (RAA) using the E-Series monitor/defibrillator (Zoll Medical Corporation, Chelmsford, MA) which provides standard biphasic defibrillation. RAA is a municipal EMS agency serving Richmond, Virginia with a population of 204,451 and a service area 62.5 square miles. RAA responds to more than 40,000 emergency calls for service (911 response) annually including approximately 225 OHCA. Patients were resuscitated using standard guidelines developed by the American Heart Association, which include combinations of chest compression, mechanical ventilation, pharmacologic therapy and electrical therapy such as defibrillation [14]. Therapeutic interventions are determined based on what the patient’s ongoing cardiac rhythm, which may change during the course of the resuscitation.

Prior to computational analysis, shocks were manually classified as either successful or unsuccessful based on the post-defibrillation ECG segments and data from the pre-hospital care record. Successful defibrillation was defined as a period of greater than 15 seconds with narrow QRS complexes under 150 beats per minute with confirmatory evidence from the medical record or ECG that a return of spontaneous circulation (ROSC) has occurred. Such evidence included lack of CPR resumption over the next minute, mention of ROSC in record, and/or rapid elevation in PetCO2 levels. While others have utilized alternative definitions that incorporate longer periods of ROSC and specific blood pressures, we chose this definition because a shorter timeframe is more clinically relevant in light of a renewed emphasis on minimizing “hands-off” time during the CPR duty cycle. [14] This short pause allows for ROSC determination and rapid return to CPR if defibrillation was unsuccessful. A total of 90countershocks were deemed usable for analysis (56 unsuccessful and 34 successful). An additional 8 countershocks were kept as prototypes for the development of RPD-PD method and not treated as part of the testing (by cross-validation) dataset.

During the study period, PetCO2 was not uniformly available or used for each resuscitation. Where available, PetCO2 data obtained from capnography (obtained from the Zoll model defibrillator) was also parsed from the subjects’ records. PetCO2 values for a total of 48 pre-defibrillation signal-segments (28 unsuccessful and 20 successful) were used to extract features that could be valuable in predicting the success of a defibrillation in terminating VF, leading to ROSC. Prediction of defibrillation success is the aim of this study.

## Methods

**Figure 1:**
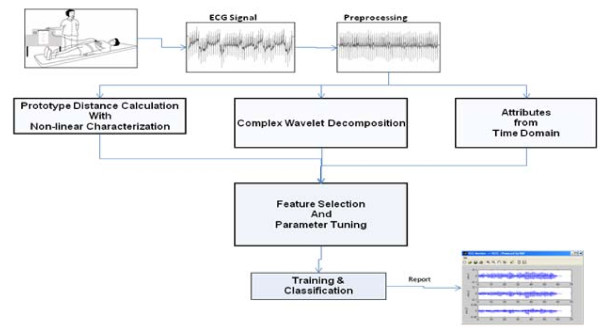
Overview of the Methodology.

Time-series features were devised in order to distinguish pre-defibrillation VF signals resulting in successful defibrillation from the unsuccessful ones. The intuitive basis for these features is further explained in subsection 3.2. We have also developed a novel non-linear method, the Recurrence Period Density Prototype Distance (RPD-PD), with stochastic recurrence periods derived from time-delay embedding. This method focuses on distributions of pseudo-periodicities while accounting for any stochasticity in the signal. Parameter selection and feature calculation for the RPD-PD model are geared toward classification (subsection 3.2). Supervised feature selection was performed to identify the most discriminative features (subsection 3.3). Selection was performed in a nested fashion so as to maintain blindness to the test folds. Simultaneous 10-fold cross-validation was used to evaluate the model. Matlab® software was utilized for all signal-processing needs. Figure 1 illustrates the high-level steps of our methodology, which is further expounded in the following three sub-sections.

### Preprocessing

The technique proposed in Shandilya et al. [15] was used to process the signals for further analyses. Some signals exhibited high frequency noise, which was attenuated by application of the Savitzky-Golay low-pass (smoothing) filter [16]. High-frequency attenuation was achieved by fitting a moving window, of width *k* data points, to a *p* ≤ *k*-1 degree polynomial by the least-squares method. For a constant *p*, *k* is set to be relatively small when only “slight” smoothing is needed; thereby making the difference between *p* and *k* to be relatively small as well. Simple averaging filters were avoided so as to better preserve the high-frequency content.

Next, sudden baseline jumps caused by interference were removed. The signal was successively ‘smoothed’ by repetitive application of Savitzky-Golay filter until only the jumps and drifts remained. The resulting signal was then subtracted from the already ‘low-passed’ signal obtained from the preceding step, yielding the cleaned signal. Frequency-domain dependent filtering methods were precluded due to the presence of all frequencies in a baseline jump and the non-stationary nature of data. Traditional high/low pass filters (such as Butterworth) cannot be employed due to spectral overlap.

### Characterization

**Figure 2:**
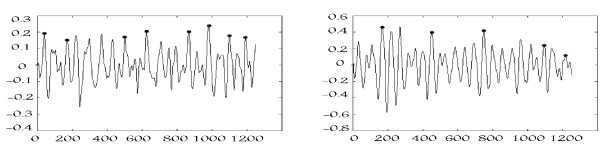
**‘Polecount’ attribute, number of peaks signified by dots, quantifies variation in the pre-shock waveforms leading to an unsuccessful shock (left) and a successful shock (right).** X-axis: samples, Y-axis: mV.

Time-series features are based on an apriori reasoning that ROSC yielding VF waveforms exhibit more activity, having properties of the coarser VF, as described above. An illustration of the Pole Count feature (Figure 2) depicts the variations in fibrillation activity of the heart along the lead II axis (sampled at 250 Hz) [15], and may at least partially represent the extent of homogeneity in VF across classes.

A dynamically adjusting threshold is used to find a minimum number of maxima, *V*_*mx*_, in the signal. The Pole-Count feature is then calculated as the number of maxima that satisfy the following condition.

(1) $$V_{\mathrm{mx}}^{i}\;>\;V_{\mathrm{mx}}^{i-1}+1.2\times \sqrt{\frac{1}{N}}\sum_{j=1}^{N}{\left(V_{\mathrm{mx}}^{i}-\bar{V_{\mathrm{mx}}}\right)}^{2}$$

Here, *V*_*mx*_ is the vector of all maxima and *N* is the length of this vector. Next, signal attributes/features are derived from the complex wavelet domain.

#### Dual-tree complex wavelet decomposition

For a signal expressed as a function of time, *t*, the wavelet transform is described by the following basis set:

(2) $$\phi_{\left(S,l\right)}\left(x\right)=2^{-S/2}\phi \left(2^{-S}x-l\right)$$

Here, *S* gives the wavelet’s width and *l* gives its position. The ‘mother function’, *Φ*, is a decaying wave-like function, altered to form the basis and subject to constraints that all members of the set are orthonormal, which provide a linearly independent set of functions. In Discrete Wavelet Transform (DWT), the scaling function, defined as follows, plays a central role in forming the basis.

(3) $$W\left(t\right)=\sum_{k=-1}^{M-2}{\left(-1\right)}^{k}c_{k+1}\phi \left(2t+k\right)$$

where *C*_*k*_‘s are the wavelet coefficients, and *k* and *M* stand for time-shift and signal length, respectively. Traditional DWT suffers from shift variance. Notably, multiple signal segments (one for each shock) are contributed by each subject. Shift variance can yield spurious features that have false correlations with outcomes. As such, the predictive model generalizes poorly, or put another way, is not discriminative. Complex Wavelet decomposition, under certain conditions, can be approximately shift-invariant without a considerable increase in computational complexity for low-dimensional signals; for our case, one-dimensional. Here, the mother function and scaling function, both have a real as well as a complex component.

(4) $$\phi_{C}\left(t\right)=\phi_{r}\left(t\right)+j\phi_{i}\left(t\right)$$

Specifically, when *Φ*_*r*_ and *Φ*_*i*_ are Hilbert transform pairs, the decomposition coefficients approach the desired shift-invariant property. This version of Complex Wavelet Transform was implemented using a ‘dual-tree’ decomposition as previously proposed [17]. Multiple attributes were then derived from the resulting coefficients at each level of decomposition, including mean, median, standard deviation, energy and entropy. Entropy was calculated as follows.

$$E=-\sum_{i=1}^{V}C_{i}\cdot \log\left(C_{i}\right)$$

Here, V is the total number of unique discrete values that the signal takes, and C is the number of times the signal takes a particular value *i*.

#### RPD-PD through Non-linear Non-deterministic time-series analysis

FT, as utilized by others [10], performs a linear transformation of a function space such that the original signal (function) is decomposed into multiple sinusoids that are globally averaged. Characterizing a short-term, non-stationary, pathological signal requires the assumptions of linearity and periodicity to be relaxed. Limitations of a Fourier based analysis have also been discussed in other studies [11,18]. As with most nonlinear time-series analyses, we begin by projecting our data *x(t)* onto a state space *p(t)*. Here, each dimension, of the state-space, itself represents a time-delay. The concept of recurrence [19] can be interpreted as measuring the level of aperiodicity in the data.

(5) $$p\left(t\right)\subset hypersphere\left(p\left(t+\delta t\right),r\right)$$

Here, the data projected onto a state-space is *p(t)*, *r* is the radius of a hypersphere defined around a state *p(n)* (where *n* is a specific value of *t*). Following the data, in state space, *δt* is the recurrence time at which data falls within the sphere, once again, after having left it. Periodicity is a special case of recurrence when *r* = 0 and all ‘states’ exhibit the same *δ*. Time delay embedding is used to project the data series into multiple dimensions of a phase space. Each dimension *m* corresponds to a multiple of the time delay *τ*.

(6) $$p_{n}=\left[p_{n},p_{n-\tau },\ldots p_{n-\left(m-1\right)\tau }\right]$$

Autocorrelation and mutual information have been suggested [19] for selecting a proper combination of dimensions m, time delay τ, and radius r. However, our objective is to separate the two classes, ‘successful’ and ‘unsuccessful’, as far as possible based on a distance metric and the given data without losing generalization power. Neither class presents apparently periodic signals. As such, the novel parameter selection regime, as proposed here, finds a ‘structure’ in the signal, defined by dimensions *m* and time delay *τ*. This structure would differ significantly in its pseudo-periodicities for the two classes. Proper parameter selection is essential in rendering this method useful. Four post-defibrillation signals that exhibited regular sustaining sinus rhythms, with narrow complexes, were selected as successful prototypes. Four defibrillation signals that induced minimal change in the ECG or were immediately followed by smooth VF after shock, with no conversion, were selected as unsuccessful prototypes. Note that selection of pre-defibrillation signals is based solely on post-defibrillation segments. Considerable variability was observed in prototypes of the unsuccessful class. Selecting more prototypes, at least for this class, should result in a better tuning of parameters (by the procedure described in next paragraph) for RPD-PD. However, this desire for more prototypes had to be balanced with the need for a relatively unbiased sample set, given the relatively small size of our dataset. Thus, the number of prototypes for this study was kept to four.

For 10-fold cross validation and a dataset with n instances, each training-set would contain *n*-(*n*/10) samples, thus leaving out the test set. A range of possible values was defined for each parameter. Recurrence period density was then calculated for each combination of parameter values and each signal in the training-set (TS) and prototype-set (PS). We define the metric *KD* (Equation 7) to calculate the pairwise distances from each TS density to all PS densities.\

(7) $$KD=\sum_{i=1}^{T}\left(1+D_{i}^{c}\right)\cdot {\left(D_{i}^{c}-D_{i}^{S}\right)}^{2}$$

Here, *s* stands for a given signal while *c* can stand for any of the other signals; *D*^*c*^_*i*_ and *D*^*s*^_*i*_ are the density values at a certain period *i*. *KD*, being inspired by the Kullback–Leibler distance, is biased towards the characteristics of *c* but, unlike KL, can also serve to measure the distance between two discrete distributions. Given classes A and B, a density from class A is subdivided into non-overlapping windows or ranges, which are compared (by *KD*) with respective windows of other densities. Therefore, our optimization is performed over a total of four variables, *m*, *τ*, *r*, and window, as follows.

Classes are maximally separated by maximizing the quantity *sep* (Equation 8). *Sep* represents closeness of all TS signals to PS signals in their own class (and remoteness from the opposite class), while also accounting for differential variation in within-class distances for the two classes. We deem this normalization necessary, as data in one class may be more homogenous than data in the other.

(8) $$sep=\sum_{i}^{L}\frac{\left(\bar{KD_{i}^{B}}-\bar{KD_{i}^{W}}\right)}{\max\left(\sqrt{\frac{1}{C^{B}}\sum_{j=1}^{C^{B}}{\left(KD_{i}^{j}-\bar{KD_{i}^{B}}\right)}^{2},\sqrt{\frac{1}{C^{W}}\sum_{j=1}^{C^{W}}{\left(KD_{i}^{j}-\bar{KD_{i}^{W}}\right)}^{2}}}\right)}$$

Here, *L* is total number of TS instances/defibrillations. For a given *i*, *KD*^*B*^ and *KD*^*W*^ are means of between-class and within-class distances, respectively, to instances in PS. *C*^*B*^ and *C*^*W*^ are total number of PS instances in the opposite class and *i*’s own class, respectively.

Each input signal from the test set is then compared to each prototype in both classes. The following distance is calculated as two features, *sKD*_*B*_ and *sKD*_*W*_, for a signal *s*.

(9) $$sKD_{B,W}=\frac{1}{Q}\sum_{p=1}^{Q}\left\{\sum_{i=1}^{T}\left(1+D_{i}^{p}\right)\cdot {\left(D_{i}^{p}-D_{i}^{S}\right)}^{2}\right\}\cdot \mathrm{sgn}\left(D^{p}-D^{S}\right)$$

Here, *Q* is total number of signals in PS for a given class, *T* is longest period in the chosen window, *D*^*P*^ and *D*^*S*^ are vectors representing densities of the prototype and *s*, respectively, and *sgn* is the sign/signum function. The average *sKD* for each class serves as an attribute of a given signal.

### Classification

**Figure 3:**
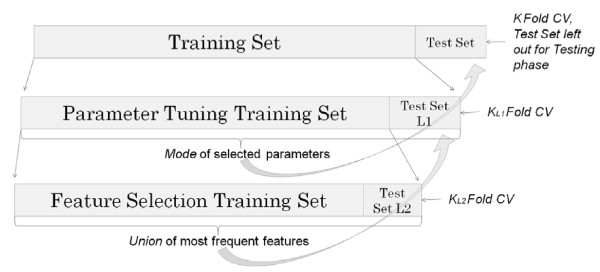
**Twice-nested cross-validation setup.** Parameter tuning is performed at Level 1 (L1), where an optimal feature subset has already been selected by cross-validation at Level 2 (L2).k = k_L1_ = k_L2_ = 10 folds; same for all levels.

Cross-Validation is frequently employed when there is a limited amount of data available. Feature selection, performed with cross-validation on the whole dataset, creates a positive bias in prediction accuracies by indirectly using information from the test set. As such, feature selection must be performed within the training set that is generated for each run of *k*-fold cross-validation. However, using the entire training set leads to over-fitting within the training set, which creates a negative bias in accuracies when the test fold is passed through the model [20]. To prevent this, and to also select parameters for the learning algorithm in a nested fashion, we employ a twice-nested version of cross-validation (Figure 3).

#### Feature selection

The feature space was searched by employing Recursive Feature Elimination (RFE) with Support Vector Machines (SVMs) [21]. For a linear SVM, the decision function is given by,

(10) $$f\left(u\right)=\sum_{k=1}^{n}w_{k}u_{k}+b$$

The weight, *w*, of each feature, *u*_*k*_, indicates the extent of each feature’s contribution to the classifier’s continuous output, and *n* in the total number of features. RFE starts by building a model with all the available features. The one with the smallest *|w|* is eliminated. At each subsequent step, the model is rebuilt and the elimination is repeated. RFE is similar to Best First Search (BFS) with a backwards approach. In contrast, by using *w*, we can reduce *n* runs to 1 run of the classifier at each step in order to eliminate the feature that leads to the smallest decrease in accuracy. Since ranking was performed with cross validation, a rank-range and rank-median was generated for each feature.

A ‘best performing’ feature subset can be defined as one that leads to the highest average (cross-validated) accuracy for a given nested cross-validation run. Traditionally, either a subset that performs best for the greatest number of nested/inner runs is chosen (thereby, partially accounting for variance or random effects in the data) or, in case where no single subset is chosen for a majority of the inner runs, a union of all chosen subsets (one for each inner run) presumably yields the best performing feature subset for the outermost test fold. Notice (in Figure 3) that two levels of nesting was used to select features *and* parameters in order to remain blind to the test fold while still being able to use cross validation for selection purposes. In order to observe variance in feature selection within the training set generated at the top most level, selection-frequencies *fs* for each feature were generated as follows.

$$fs=\frac{S_{L2}}{k_{L1}\cdot k_{L2}}$$

Here, S_L2_ is the number of all inner runs at level 2 (see Figure 3) for which the feature was selected. k_L1_ and k_L2_ are the number of cross-validation folds at level 1 and level 2, respectively. These frequencies showed that 3 to 5 features were selected for only 20% of the innermost runs, indicating some further room for reduction in model variance by elimination of these spurious features. As an alternative to the traditional “wrapper” approach [20], we formulate a new data matrix with features that were found to be members of the best-performing feature-subsets for at least 70% of the runs. This new approach (Figure 3 Level 2) boosted accuracy by approximately 3% without violating blindness to the outermost test folds. Furthermore, at level 1, the combination of parameters that was selected most often for the k = 10 test folds, i.e. *mode* of the selected combinations, was used for final classification of instances in the outermost test fold. The underlying cost-sensitive regime responsible for selecting features for any given training set is as follows.

As our dataset is imbalanced, with unsuccessful to successful ratio of about 2 to 1, so classification must be cost-sensitive. A cost insensitive approach upstream, i.e. feature selection, may preclude some features that would contribute to a decision boundary strictly between the two classes. In the absence of such features, even cost-sensitive classification yields a decision boundary that is drawn to maximize accuracy only. In order to compensate, false negatives were penalized twice as much as false positives. In other words, feature ranking through RFE-SVMs was done with a 2:1 cost of misclassification.

Time-series and complex wavelet features were also extracted from the PetCO2 signal using the exact same methodology as for ECG signals.

**Figure 4:**
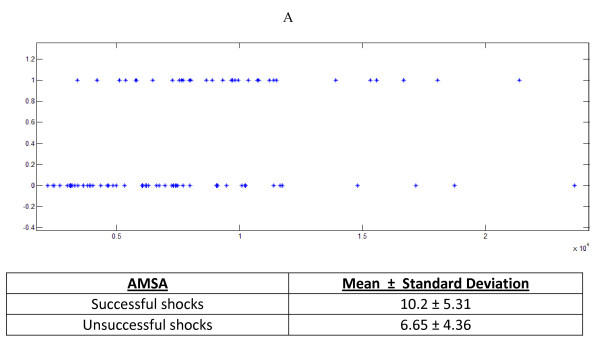
**AMSA feature (A) Instances/Shocks are plotted against classes ‘0’(unsuccessful) and ‘1’(successful).** No clear threshold can be identified for separating the classes. (B) Means and Standard Deviations present significant overlap.

We have compared our ECG-only based method to the AMSA method [10], which decomposes ECG signals with FT. AMSA is calculated as the sum of frequencies weighted by their amplitudes. We replicated the procedure to calculate AMSA and tried to discern a threshold (see Results and Figure 4). ROC analysis was used to evaluate reliability of all models by calculating area under the curve (AUC). Accuracy was calculated as the average percentage, over all cross-validation runs, of instances that were correctly classified. All accuracy, sensitivity and specificity values are reported for the best decision threshold found for the given test and/or algorithm (see Results).

SVM was preferred as the general machine learning framework for classification over structures such as neural networks and radial basis function networks, primarily because of studies that have shown that when limited amount of training data is available, neural networks [22] and radial basis functions [23] may not provide desirable generalization performance and may overfit the data.

## Results

**Figure 5:**
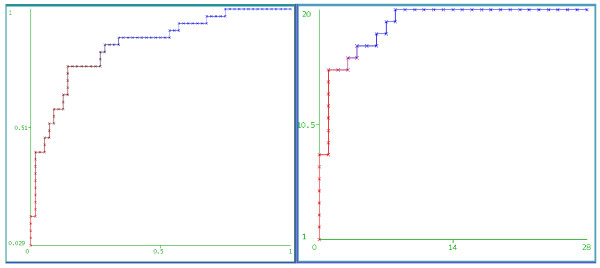
**Receiver operating characteristic curves (A) for a model built using all 90 shocks and ECG signal only, and (B) for a model built using 48 shocks and ECG + PetCO2.** (**A**) X-axis::1-Specificity, Y-axis::Sensitivity. (**B**) X-axis::False Positives, Y-axis::True Positives. Threshold ranges from 0 to 1 as color transitions from pure blue to pure orange.

Classification using our machine-learning approach with 6–10 features yielded an ROC AUC of 85% and accuracy of 82.2%, for the model built with ECG data only (Figure 5). Integrating PetCO2 features boosted ROC AUC and Accuracy to 93.8% and 83.3%, respectively, for a total of 48 shocks with usable PetCO2 segments. A large ROC AUC allowed for 90% Sensitivity and 78.6% Specificity at a classifier-output threshold value of 0.22. Classifier (support vector machine: SVM) output for each instance is compared to this value before it is assigned to a class. For classification problems, varying this threshold is a common way to assign more weight to one class than the other. As only a limited number (48) of usable PetCO2 signals were available, these results will need to be confirmed on larger datasets.

Using the methodology proposed by Ristagno and colleagues [10], no clear AMSA threshold could be identified (Figure 4) to distinguish successful shocks from unsuccessful ones. Employing a C4.5 [24] based decision stump or 1-rule for AMSA values yielded 44.1% Sensitivity and 77.2% Specificity. ROC AUC for AMSA was 60.9%. C4.5 is one of the first-introduced and most commonly used machine learning methods which creates subsets from a given sample set by minimizing entropy of the samples’ class membership within the resulting subsets. It is a common and efficient way of creating a 1-rule where a threshold is not apparent by visual inspection. PetCO2 data was not used in the examination of AMSA.

**Figure 6:**
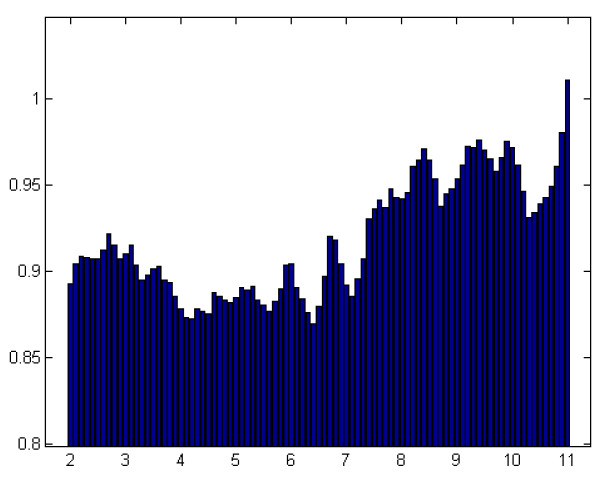
**Bar plot of information content, measured by*sep,*as a function of signal duration in seconds (x-axis).**

Pre-shock signal length may also be optimized to provide maximum information content, and thus better discriminating features. In order to visualize how information content changes with signal duration, the signal’s window size is incremented from 2 seconds to 11 seconds with 0.1 second steps. Separation along each dimension of the feature space is calculated by equation (8) and the mean of the top 5 most discriminating dimensions is plotted (Figure 6). As a heuristic, we consider a separation of less than 0.8 (*sep* < 0.8) to be non-discriminative.

## Discussion

Once VF has transitioned into the mother rotor form [7], defibrillation should occur as soon as possible. Passage of time, in any pulseless rhythm, is the most significant of survival determinants [9,25]. Effects of VF duration, which may or may not be countered by CPR, may be a pre-determining factor for defibrillation outcome. Many previous studies have aimed to quantify VF duration. The focus, instead, should be on improving the probability of ROSC as CPR is delivered, thereby directly targeting and identifying features that are related to outcome. Such an approach will also be effective in identifying treatments that will maximize chances of ROSC if they can be linked to improving the signal. While it could be argued that an additional goal of the method should be to predict return of an organized rhythm (ROR) as opposed to ROSC, doing so may not improve performance since ROR without ROSC is essentially PEA and is associated with worse outcomes. However, the ability to distinguish the two may provide insight into developing new treatments and understanding of cardiac arrest.

Previous studies [11,12,26] have established the advantages of a ‘wavelet’ approach over FT in evaluation of VF. However, their definitions of shock success are similar to that of Ristagno and colleagues [10]. In order to overcome limitations such as the shift variance of traditional DWT, we report a first-use of Complex Wavelet decomposition designed for defibrillation outcome prediction (and for any ECG analysis). Additionally, instead of quantifying the presumably varying degree of aperiodicity across classes through time-delay embedding [27], RPD-PD separates distributions of frequency content; thereby distinguishing two signals that differ in more ways than just perceived ‘randomness’.

Whenever cross-validation is employed with feature selection or parameter tuning, a twice-nested implementation is requisite for obtaining results that are unbiased by information in the test set. This follows from the assumption that field application will produce previously unseen data, providing a true test for the model. Additionally, there is usually a tradeoff between complexity of the predictive model and its generalization power. As complexity is partly defined by the number of features and values of the machine learning algorithm parameters, nested cross-validation also provides a way to optimize this tradeoff.

While the number of subjects with usable PetCO2 values was small, the addition of PetCO2 to the algorithm appears to significantly improve performance. This is not surprising given the positive correlation between PetCO2, cardiac output, and coronary perfusion pressure produced during CPR [28,29].

### Limitations and future work

Larger datasets, of 5–10 times the size of our current dataset, will be required to further test the model. We anticipate significant improvements in performance as the feature space becomes more densely populated and additional physiologic signals are added. Development of prediction techniques using multiple signals may provide the greatest value if the value of each signal is understood. This is important since, depending on the clinical system, each signal may not be clinically available for use by health care providers.

Certainly, controversy will exist regarding the definition of successful defibrillation. While linking the definition with longer-term patient outcomes is attractive, in reality, these outcomes are dependent on several variable factors. Such factors include the use of antiarrhythmics among paramedic systems, the amount of vasopressors used during the resuscitation, the underlying cause of the arrest, and even interventions such as induction of hypothermia intra-arrest and comprehensive post-resuscitation care. For these reasons, we believe that our definition of successful defibrillation will serve future studies well.

## Conclusions

We have developed a novel algorithm for predicting successful defibrillation of VF. The model is built upon knowledge extracted with multiple signal-processing and machine-learning methods. The proposed ECG characterization, combined with information extracted from PetCO2 signals, shows viability for decision-support in clinical settings. Our approach, which has focused on integration of multiple features through machine learning techniques, suits well to inclusion of multiple physiologic signals.

Based on the results obtained, we can also draw confidence in our hypothesis that random effects, as proved by Gundersen and colleagues [13], can be countered by inclusion of multiple physiological signals. Success of an integrative, information-theoretic approach should bode well for the field of defibrillation outcome prediction, which suffers from low specificities.

## Competing interests

Sharad Shandilya, Kevin Ward and Kayvan Najarian.

Disclosure: Patent pending subject to Virginia Commonwealth University.

## Authors’ contributions

KW and MK provided medical domain expertise, clinical data analysis, and manuscript editing. KN provided technical expertise for computational data analysis, and manuscript editing. SS provided technical expertise, computational data analysis, model development, and drafted the manuscript. The authors wish to thank Zoll Medical Corporation for their technical assistance in accessing information from the cardiac arrest database. All authors read and approved the final manuscript.

## Pre-publication history

The pre-publication history for this paper can be accessed here:

http://www.biomedcentral.com/1472-6947/12/116/prepub
